# Disproportionate Declines in Ground-Foraging Insectivorous Birds after Mistletoe Removal

**DOI:** 10.1371/journal.pone.0142992

**Published:** 2015-12-07

**Authors:** David M. Watson

**Affiliations:** Institute for Land, Water and Society, Charles Sturt University, Albury, NSW, Australia; Centre for Cellular and Molecular Biology, INDIA

## Abstract

Insectivorous birds have been recognized as disproportionately sensitive to land-use intensification and habitat loss, with those species feeding primarily on the ground exhibiting some of the most dramatic declines. Altered litter inputs and availability of epigeic arthropods have been suggested to underlie reduced abundances and shrinking distributions but direct evidence is lacking. I used a patch-scale removal experiment in southern Australia to evaluate whether ground-feeding insectivores are especially vulnerable to altered litter-fall. Building on work demonstrating the importance of mistletoe litter to nutrient dynamics, litter was reduced by removing mistletoe (Loranthaceae) from one set of eucalypt woodlands, responses of birds three years after mistletoe removal compared with otherwise similar control woodlands containing mistletoe. Despite not feeding on mistletoes directly, insectivores exhibited the greatest response to mistletoe removal. Among woodland residents, ground-foraging insectivores showed the most dramatic response; treatment woodlands losing an average of 37.4% of their pre-treatment species richness. Once these 19 species of ground-foraging insectivores were excluded, remaining woodland species showed no significant effect of mistletoe removal. This response reflects greater initial losses in treatment woodlands during the study (which coincided with a severe drought) and double the number of species returning to control woodlands (where mistletoe numbers and litter were not manipulated) post-drought. These findings support the productivity-based explanation of declining insectivores, suggesting diminished litter-fall reduced habitat quality for these birds via decreased availability of their preferred prey. In addition to altered prey availability, interactions between litter-fall and epigeic arthropods exemplify the importance of below-ground / above-ground linkages driving ecosystem function.

## Introduction

Studies of woodlands and forests in many parts of the world are reaching a recurring conclusion: insectivorous birds are in trouble. Whether in the hedgerows of Europe [1], prairies of North America [2] or rainforests of southern Asia [3], formerly widespread birds which consume arthropods are declining [4, 5]. Rather than all insectivores, those species that feed primarily on the ground consistently display greater sensitivity than other groups, especially in areas experiencing land-use intensification [4, 6]. While some of these species forage upon coarse woody debris or within understorey plants, many feed primarily on the forest floor [7, 8]. Rather than already rare species becoming rarer, many of these reported declines involve abundant species [9, 10], suggesting ecosystem-wide consequences may arise as these losses reverberate across food webs.

Various factors have been implicated in declines of insectivores [4, 11, 12], but an emerging hypothesis suggests bottom-up mechanisms mediated via altered litter-fall may be crucial ([13], see also [14]). Selective habitat loss on more productive soils combined with reduced densities of Nitrogen-fixing understorey shrubs (through initial clearing and subsequent browsing by domestic stock) and altered flows of both nutrients and water due to land-use intensification have fundamentally altered the foundation of woodland and forest food webs [13]. Applying this productivity-based hypothesis to woodland bird declines [7], I suggested sites with greater leaf litter supported more epigeic invertebrates enabling higher abundance, species richness and temporal stability of woodland bird assemblages, with ground-foraging insectivores exhibiting the greatest responses.

Mistletoes and other parasitic plants are important sources of litter-fall [15, 16], shedding large volumes of enriched litter in discrete patches, thereby increasing heterogeneity in resource availability [17, 18]. Despite representing 3–4% of above-ground biomass, the hemiparasitic herb *Bartsia alpina* was found to generate ~17% of annual litter-fall in a subarctic heathland, delivering a 53% increase in N input and accelerating total litter decomposition by 21% ([19], see also [15]). Most research on parasitic plant litter has focused on changes to nutrient availability [20], plant growth [21] and understorey plant diversity [22], but effects on soil microbial communities [23], epigeic arthropods [24] and successional dynamics [25] have been reported. Collectively, this work informs a growing awareness of the role that parasitic plants play as facilitators, particularly in low productivity systems [26, 27].

Using a manipulative experiment of mistletoe at the woodland scale [28], relationships between resource availability and species occurrence can be disentangled. In this contribution, I address the specific question “Are ground-foraging insectivores more sensitive to reductions in leaf litter than other bird groups?” using a before/after control/impact experimental design. An assessment of how resource availability interacts with patch and landscape-scale factors will be explored elsewhere. This question relates to predictions 3 and 5 of [7]: those sites with greater litter-fall expected to have higher species richnesses and abundances of ground-foraging insectivores, and exhibit lower species turnover (within and between years). Unlike trees or understorey plants, it is feasible to remove mistletoe at the woodland scale without disturbing the soil or altering other aspects of habitat structure. Since mistletoe represents a major source of litter in eucalypt woodlands (almost doubling total litter-fall [17]), litter-fall can thus be manipulated at the woodland scale, enabling effects on woodland birds to be discerned. This study coincided with a prolonged drought affecting southern Australia, thereby quantifying the influence of litter-fall on patch-scale distribution of birds during a period of heightened scarcity where determinants of distribution might be expected to be more clear-cut ([10], and references therein]).

## Materials and Methods

### Study area and experimental treatment

All sampling was carried out under Scientific Research Permit S10921 issued by the then Department of Environment and Climate Change (Parks and Wildlife Division) and the authority of Charles Sturt University Animal Care and Ethics Committee approval 05/054. This experiment was conducted in the upper Billabong Creek catchment in southern New South Wales, Australia (35°42 S 147°19E). Forty woodland remnants were used as study sites, isolated 80–100 years ago when surrounding habitats were cleared for agriculture. Sites ranged in area from 1.3–23.9 ha (mean of 9.8) with tree cover in the surrounding countryside ranging from 2.5–52.2% (mean of 19.7%; calculated from circles of 1 km radius centred on each site). The ground layer of all sites was dominated by perennial grasses with occasional shrubs (*Acacia* spp., *Exocarpus* spp.) in low densities. Mistletoe—principally *Amyema miquelii* (Loranthaceae), a eucalypt parasite that forms dense pendulous clumps at the edge of the canopy—occurred at medium-to-low densities in these woodlands and also infected scattered trees in surrounding agricultural land and roadside stands (see [28] for further details about study area and site selection). The study (2003–2008) coincided with a prolonged drought affecting southern Australia between 2000 and 2010, representing the period of lowest rainfall in the region since records began in 1900 [29]. Annual rainfall totals recorded at the two closest weather stations (Albury and Wagga Wagga) were less than the long term average for six of the eight years between 2001 and 2008 (Bureau of Meteorology data).

Having completed one year of pre-treatment bird surveys in 2003/2004, treatment (patch-scale mistletoe removal) was assigned to 20 woodlands using a blind geostratified-random approach. Treatment and control groups spanned the same ranges of patch area, vegetation structure and landscape context, and had comparable pre-treatment mistletoe density and species richness of birds [28] allowing strong inference about the direct effect of resource reduction on bird numbers. Although selected to minimize variation in overall vegetation type, these woodlands varied considerably in management history, area and degree of isolation from other remnant vegetation. To minimize the confounding effects of this variation on the outcomes of experiment, resource-reduction effects were considered in terms of proportional rather than absolute change.

Mistletoe plants growing within treatment woodlands were systematically removed in 2004 using extendable loppers and pole saws from trailer-mounted hydraulic boom-lifts. Mistletoe plants and associated sections of host branches were left where they fell—directly beneath their former hosts. Sham removals were conducted in the canopies of control sites, driving within the woodland using the same equipment and removing branches from infected and non-infected trees but leaving all mistletoe plants intact. As with treatment sites, no material was removed from control sites—all pruned branches were left where they fell. Some woodlands did not contain mistletoe, resulting in three groups: control woodlands with mistletoe (n = 11), treatment patches with mistletoe plants removed (n = 17) and woodlands from which mistletoe was naturally absent (n = 12). To measure the direct effect of litter reduction resulting from the mistletoe removal experiment, all analyses included here relate solely to comparisons between control and treatment woodlands (n = 28; see [28] for further details of study design).

### Bird surveys

To estimate bird species richness in these woodlands, inventories were compiled from patch-scale surveys conducted in all four seasons during two year-long periods: prior to treatment (April 2003 through February 2004) and three years after treatment (April 2007 through February 2008), providing 8 surveys total per woodland. Surveys were conducted using the standardized search [30], applying a quantitative results-based stopping rule to determine the number of 20 min samples per survey. The stopping rule employed—‘stop sampling once recorded richness of woodland-dependent species exceeds 80% of predicted richness’—used the Chao2 estimator [31] and yielded seasonal surveys of 3–6 samples (*i*.*e*., efforts of 60–120 min) of ≥ 80% completeness; 260 of the 320 surveys tripping the rule after the minimum three samples (i.e., the modal survey duration was 60 min). Comprehensive comparisons of various methods and efforts [32] demonstrated the dynamic avian assemblages inhabiting these small woodlands required greater efforts than the 10 or 20 min surveys typically used to sample birds in habitat fragments. Moreover, as resource manipulation was conducted at the woodland scale, results-based stopping rules enabled sampling to be carried out at the woodland scale while still generating comparable richness estimates. By scaling sampling effort to estimated completeness rather than using fixed effort approaches, the confounding effects of any inter-specific or patch-level differences in detectability (including treatment effects) were avoided and overall survey completeness standardized. Sampling involved walking throughout the woodland and identifying all birds seen or heard within the woodland, including species flying beneath the canopy. Sampling was conducted only during favourable weather conditions, avoiding periods of heavy rain, strong wind or intense heat, primarily in the four hours after dawn and three hours before dusk, but continuing throughout the day if conditions were favourable.

In addition to yielding species richness estimates of comparable completeness, this approach generated incidence measures for each species in each woodland remnant, expressed as the proportion of samples in which it was detected [30]. Thus, if a particular woodland required four samples for each of four seasonal surveys and a particular species was recorded as present during eight of those samples, the incidence was 0.5 (8/16). Given concerns about variation in detectability, abundances were not estimated, but a recent comparison in similar habitat demonstrated a close relationship (r = 0.88) between incidence and median density estimates for forest fragments [33], with incidence less sensitive to the confounding effects of variation in detectability [30]. As well as species-specific values, summed incidences were calculated for functional groups by adding the values for each species in each treatment group (e.g., total insectivores in control woodlands). An additional richness measure was calculated—‘Resident Richness’: those species recorded in at least two seasons for the given year, excluding transients detected in a single season.

### Data manipulation and analysis

Five subsets of these three variables (summed incidence, species richness and resident richness) were distinguished for each woodland patch and analysed separately. ‘Insectivores’ (including open country species, aerial foragers and woodland-dependent species) were defined as any species depending on arthropods as a principal food source (74 species, defined using species-trait database compiled by [8]) and ‘Everything Else’; species reliant on all other nutritional resources (53 species;). “Woodland Birds” were defined as species dependent on woodland as their primary habitat (excluding open country species, aerial foragers and exotic species (75 species), with two subsets of woodland birds compared against one another: ‘Ground Foragers’: woodland-dependent ground-foraging insectivores (19 species that forage for insects primarily on the forest floor, after [8] and ‘Other Woodland Birds’; remaining woodland birds reliant on other nutritional resources, comprising 15 species of granivores, nectarivores and fruguivores; and 41 species of insectivore foraging on arthropods elsewhere (56 species in total). Responses to resource reduction (via patch-scale mistletoe removal) were measured in terms of change between pre- and post-treatment inventories, *i*.*e*., the datum for year 1 (2003/2004) subtracted from the equivalent datum for year 2 (2007/2008) expressed as a proportion of the year 1 (pre-treatment) value.

Pre- and post-treatment incidences of each woodland-dependent species were compared and classified into one of five categories for each woodland: ‘Colonization’ (absent in year 1, present in year 2); ‘Local extinction’ (present in year 1, absent in year 2);’Increaser’ (incidence higher in year 2); ‘Decreaser’ (incidence lower in year 2); and no change. No cut-off was used in these determinations as there was no *a priori* means of distinguishing a significant change from background fluctuation. Treatment sites were compared to control sites in terms of the proportion of the avifauna in these categories as well as two composite variables: ‘Net Increase’ (= Colonizations + Increasers) and ‘Net Decrease’ (Local extinctions + Decreasers), with additional comparisons conducted between ground foragers and all other birds. Since these proportions are expressed relative to the year 1 datum, the value for colonizations during the study period pushed totals for each woodland / group beyond 1.0. Prior to comparing means (two-tailed test, using either Student’s *t* or Mann-Whitney *U* as the test statistic and P = 0.05 as the significance threshold), groups were checked for equality of variances using Levene’s test, adjusted degrees of freedom used where heteroscedasticity was detected.

To complement these richness and incidence-based metrics, occurrence data were compiled for both woodland-dependent species and ground-foraging insectivores; changes in patch occupancy compared between treatment and control woodlands. Based on the ratio of species colonizing or becoming locally extinct during the study, woodlands were classified as either ‘Local extinction dominant’ (local extinctions exceeded the number of colonists), ‘Colonization dominant’ (colonists exceeded local extinctions) or ‘No net change’, differences tested using Fisher’s Exact Test (significance threshold set at P = 0.05).

## Results

### Effects of resource reduction on insectivore incidence

**Fig 1:**
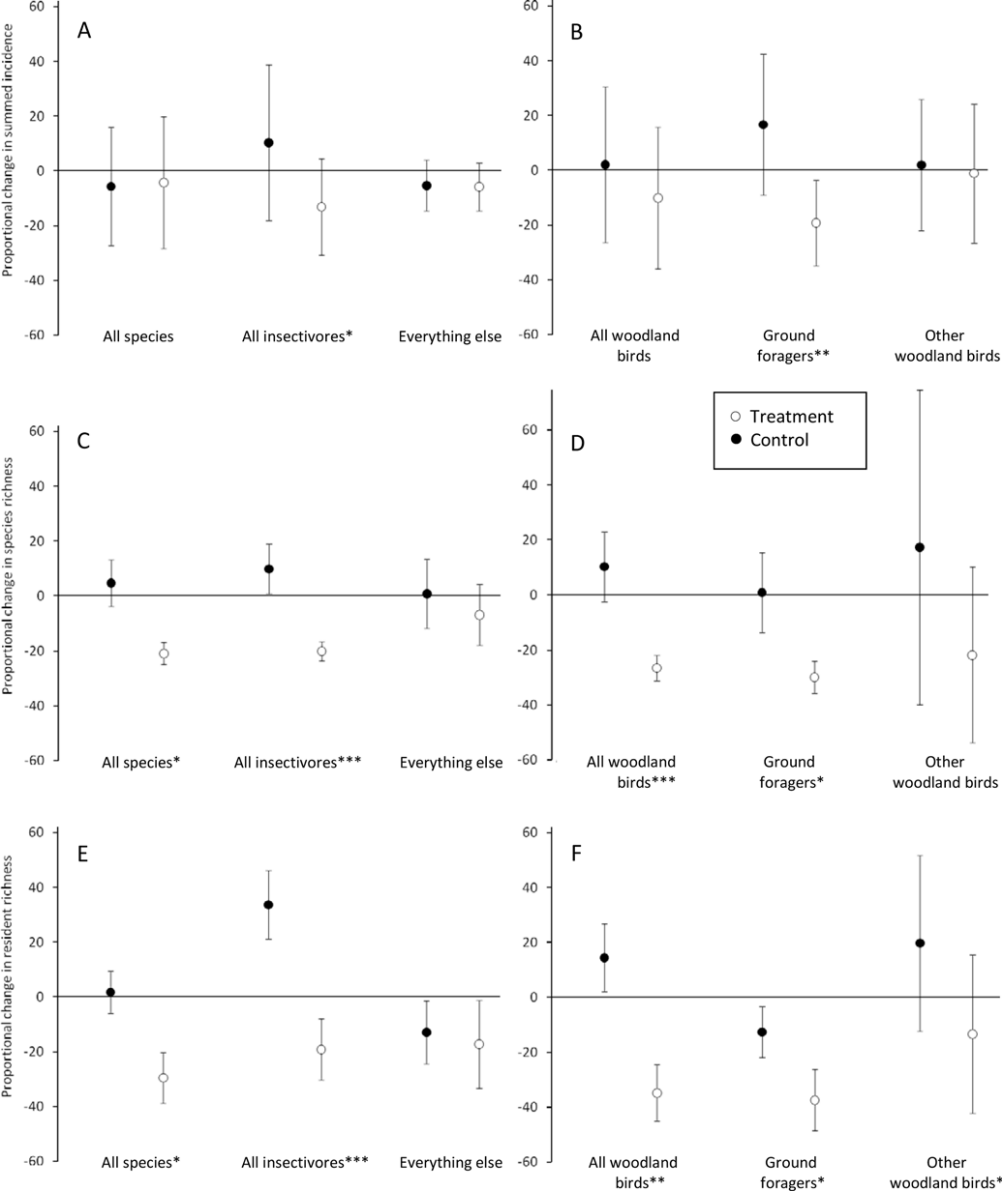
Plots depicting changes in bird occurrence associated with litter reduction at the patch scale via experimental removal of mistletoe. Closed circles are means for the 11 control woodlands, open circles are means for the 17 treatment woodlands and error bars represent one standard deviation of the mean. All plots are proportional, changes in bird occurrence expressed as a function of the initial (pre-treatment) value. Three variables are evaluated—summed incidence (A, B), species richness (C, D) and resident richness (number of bird species recorded in woodlands in at least two seasons per year; E, F). Left hand plots (A, C, E) relate to all bird species recorded within the woodland fragments, distinguishing those species feeding on insects from species reliant on other foods; right hand plots (B, D, F) depict woodland-dependant birds, distinguishing ground-foraging insectivores from other woodland species (including insectivores foraging in other strata). Means of control and treatment woodlands were compared using Mann Whitney U tests (two-tailed), one asterisk for p <0.05, two for p<0.001, three for < 0.001.

As reported previously [28], mistletoe removal resulted in dramatic changes to bird occurrence; treatment sites lost an average of 20.9% of their original number of species, 26.5% of woodland-dependent species and 34.8% of woodland-dependent residents (compared with increases of 4.7%, 10.2% and 14.5% in control woodlands, respectively). Using those assemblage-wide analyses as the starting point, overall responses of bird assemblages to resource reduction were partitioned into those changes exhibited by insectivores and those changes shown by all other groups combined. Using summed incidences from the four seasonal bird surveys per woodland per year, there was no discernible difference in proportional change between control and treatment woodlands for all species combined (Fig 1A). In contrast, total insectivore incidence declined by 13.1% (SD = 17.6) in the treatment woodlands and increased by 10.3% (SD = 28.4) in the control woodlands (U = 49.0; P = 0.036). All remaining species (*i*.*e*., those dependent on foods other than insects) showed a similar trend to the overall pattern, slight declines in incidence in both treatment and control woodlands, but not differing significantly from one another. Restricting analysis to those species dependent on woodland as their primary habitat, the influence of ground-foraging insectivores became apparent, declining by 19.2% (SD = 15.6) in woodlands where mistletoe was removed but increasing by 16.7% (SD = 25.7) where mistletoes remained. Once this foraging guild was removed, the remaining assemblage of other woodland birds showed no effect of mistletoe removal (Fig 1B).

### Effects of mistletoe removal on insectivore richness

Insectivores accounted for the overall decline in species richness (Fig 1C), decreasing by 20.0% (SD = 3.5) post treatment while increasing by 9.8% (SD = 9.1) in control woodlands. Declines of ground-foraging insectivores were more marked, with a mean proportional decline of 29.9% (SD = 5.8; Fig 1D).

To discern effects of resource reduction on woodland residents, those transient species recorded in a single seasonal survey in each woodland were excluded. Treatment woodlands lost 29.5% (SD = 9.3) of their pre-treatment assemblage of resident species and, although resident insectivores declined by 19.2% (SD = 11.2) they exhibited a 33.6% (SD = 12.5) increase in control sites (U = 35.5; P = 0.005; Fig 1E). Once insectivores were excluded, the remaining assemblage showed no treatment effects. Restricting analysis to woodland-dependent residents, overall declines were observed in all three groups examined but were most pronounced in the ground-foraging insectivores (mean of 37.4%, SD = 11.1). Differences were also detected in the control sites (Fig 1F)—total woodland residents and non ground-foraging residents both increased (by 14.5% (SD = 12.3) and 19.8% (SD = 32.1) respectively), while ground-foraging insectivores declined by 12.6% (SD = 9.3).

### Effects of mistletoe removal on temporal variation in community composition

Temporal turnover was quantified at the woodland scale by comparing the proportion of species gained or lost between the pre and post-treatment years (Table 1). The three differences deemed statistically significant between treatment and control sites all relate to increases in control woodlands rather than decreases in treatment sites. Thus, control woodlands gained an additional 47.7% of the number of woodland species recorded pre-treatment, compared to a 21.6% increase in the 17 treatment woodlands (t = 2.21, P = 0.049, adjusted d.f. = 17.8). When these colonizing species are added to the number of species that increased in incidence the proportion climbs to 75% for the 11 control woodlands compared to 41.9% for the 17 treatment patches (t = 2.41, p = 0.032, adjusted d.f. = 11.3). The same comparisons restricted to those 19 species of ground-foraging insectivore yielded congruent results; the proportion of colonists in control sites more than double the value for treatment woodlands, a statistically significant difference (t = 2.47, P = 0.02, adjusted d.f = 13.8). In terms of occurrence at the patch scale, the same patterns were evident (Table 1), treatment woodlands experiencing significantly greater local extinctions than control woodlands for woodland dependent species (Fishers Exact Test; P = 0.0087), changes in ground-foraging insectivores showing the same pattern, but not significantly different (Fishers Exact Test; P = 0.22).

**Table 1:** Changes post-treatment in woodland birds in mean incidence (proportional change in summed occurrences across four seasons pre vs. post-treatment), and number of sites showing specific changes in incidence.

		Woodland-dependent species		Ground-foraging insectivores	
		Control	Treatment	Control	Treatment
Mean incidence	Local extinctions	0.394 (0.091)	0.481 (0.159)	0.303 (0.194)	0.393 (0.279)
(proportion)	Decreasers	0.303 (0.116)	0.271 (0.108)	0.198 (0.117)	0.301 (0.361)
	Net Decrease	0.697 (0.163)	0.752 (0.105)	0.501(0.222)	0.693 (0.411)
	Colonizations	**0.477 (0.384)**	**0.216 (0.088)**	**0.326 (0.222)**	**0.159 (0.135)**
	Increasers	0.273 (0.142)	0.203 (0.078)	0.163 (0.141)	0.176 (0.206)
	Net Increase	**0.750 (0.436)**	**0.419 (0.137)**	0.490 (0.233)	0.336 (0.253)
N sites	Local extinction dominant	**5**	**15**	6	12
	Colonization dominant	**6**	**1**	5	3
	No net change	0	1	0	2

Summary of changes in bird occurrence across the 11 control and 17 treatment woodlands pre-treatment (2003/2004) and post-treatment (2007/2008). The first six rows denote changes in incidence (mean and standard deviations) for each group of woodlands (11 control woodlands, 17 treatment woodlands); the bottom three rows denote numbers of woodlands in each category. Incidence (the proportion of samples in which a species was detected) for each species (of the 75 woodland-dependent bird species and the subset of 19 ground-foraging insectivores) values were summed and expressed as mean proportions (and standard deviation) of the pre-treatment value: ‘Local extinctions’ were species recorded in pre-treatment but not post-treatment, ‘Decreasers’ exhibited a lower incidence post-treatment first and ‘Net decrease’ is the sum of the former two; ‘Colonizations’ were birds only recorded post-treatment, ‘Increasers’ exhibited higher incidences post-treatment, and ‘Net Increase” is the sum of the former two. Comparison of mean tests (t-tests, adjusted if Levene’s test revealed significant differences in variances) were conducted between treatment and control groups; significant (p < 0.05) comparisons denoted in bold. Occurrence data (lower three rows) is summarised for each woodland, distinguishing those woodlands that lost species during the study (local extinction dominant), gained species during the study (colonization dominant) or exhibited no net change, separate values compiled for Woodland-dependent species and Ground-foraging insectivores. Fisher’s Exact tests were conducted for the two groups separately, significant (p < 0.05) differences denoted in bold.

Considering these overall changes in occurrence individually for those 19 species of ground-foraging woodland insectivore, 11 species displayed overall decreases in the number of woodlands occupied, seven exhibited overall increases, and one was unchanged between years. In the 17 treatment woodlands, 12 species decreased and five increased patch occupancy (one species was absent, one was unchanged); whereas in the 11 control woodlands, six species decreased, six increased and there was no change in patch occupancy of a further three species (four species were absent).

## Discussion

Rather than a community-wide response to mistletoe removal, these analyses distinguish those birds foraging for insects on the forest floor as disproportionately sensitive to resource reduction, losing 37% of the initial number of resident species. Indeed, once these 19 species were excluded, all remaining woodland species (including canopy insectivores, frugivores, nectarivores and seed eaters) showed no significant effect of mistletoe removal. Although all insectivores consistently increased in control sites over the course of this study, ground-foraging insectivores decreased in both treatment and control woodlands. This difference was most apparent in the woodland residents: all insectivores increasing by more than a third but ground-foraging insectivores decreasing by 12.6%. Thus, in addition to pronounced responses to mistletoe removal, these ground foraging insectivores were declining regardless—decreases in the number of woodlands occupied were recorded for 11 of the 19 species over the course of this study.

### Dynamism of bird assemblages

Consistent with previous work on avian occurrence patterns in southern Australia [34, 35] assemblages were dynamic over time, many species coming and going from individual woodland fragments during the six-year study. The sites used for this study were small and many were in relatively poor condition (heavily grazed with little regeneration and scant coarse woody debris) and were therefore unlikely to support resident breeders of many woodland-dependent species [13]. Their small size, coupled with the extensive efforts used to survey birds at the whole-of-patch scale and the open condition of the agriculture-dominated landscapes within which these woodlands occur mean that I can be confident that reported dynamics in the bird assemblage relate to underlying changes in occurrence patterns rather than sampling artefacts. Thus, independent of mistletoe removal, 39% of the woodland birds recorded in control woodlands the first year were not recorded in the second, with 48% showing the reverse pattern. The study period coincided with the most prolonged and severe drought recorded for southern Australia [29]. In addition, there was a long-term downward trend in January–June rainfall, previously found to be the most important factor determining local abundances of insectivorous birds ([10], see also [11]). The direction and magnitude of the individual species patterns reported here are consistent with movement into the study region from regions more severely impacted by the drought to the north and west as well as the redistribution of individuals from populations residing in some of the very large, intact woodlands nearby.

### Influence of mistletoe on habitat quality

Examining the interaction between these regional patterns and the effect of resource reduction, the dramatic decline of insectivores stemmed from a reduced capacity to rebound rather than disproportionate initial losses. Indeed, considered individually, insectivore species experienced proportionally fewer declines than all species of woodland birds combined, in terms of both decreased frequency of occurrence as well as increased likelihood of local extinction (Table 1). Rather than greater losses, the effect of mistletoe removal on resource availability within woodlands led to dramatic reductions in the rate of return of woodland birds. This difference was especially pronounced in the immigrants (those species that were not recorded in a woodland in year 1, but colonized over the course of the study), proportions for both woodland-dependent species and ground foraging insectivores in woodlands with mistletoe found to be more than double the proportion in those woodlands where mistletoe had been removed.

These findings provide strong support for the predicted role of mistletoe as a nutritional subsidy during periods of regional scarcity, areas with mistletoe experimentally-removed predicted to have “communities with increased sensitivity to drought and other rare events” ([36], p. 239). Thus, these parasitic plants provide sufficient resources to allow small and otherwise poor-quality habitats to support and maintain species declining elsewhere. These patch-scale and species-level responses provide strong support for the ‘Dryad’ hypothesis, whereby mistletoe and parasitic plants generally are regarded as facilitators in low productivity habitats, boosting heterogeneity in nutrient availability and productivity by shedding large quantities of enriched leaf litter [26]. While effects on soil-based processes and understorey plants have been documented elsewhere [15, 27], this mechanism also explains why ground-foraging insectivores consistently displayed the greatest response. Recent work has demonstrated higher diversities and abundances of arthropods beneath trees infected by mistletoe ([24], Medallo-García and Hobby, unpublished data). Whether the recorded response of ground-foraging insectivores relates to changes in prey abundance, accessibility or nutritional quality is unclear [37, 38], with further work need to address this gap and determine the influence of nutritional requirements on patch occupancy and distributional trajectories at regional scales [39].

### Litter and litter dwelling arthropods as critical resources

The sensitivity of epigeic arthropods to litter depth and drought has previously been documented, different groups either leaving, dying or entering quiescent stages until more favourable conditions return [40]. Thus, I suspect the changes in insectivore occurrence detected relate to both accessibility and abundance of prey, especially in winter-rainfall dominated regions where food availability is already scarce during hot dry summers [7]. While manifested in ground-foraging birds, numerous other groups of ground-feeding insectivores rely on epigeic arthropods for nutrition, their lower vagility and increased aversion to gap crossing combining to make declines toward local extinction even more likely [13, 41].

Whereas these findings are consistent with reduced food availability for insectivores mediated via changed to litter-fall, alternative interpretations of these data are worth considering. The experimental treatment may have had an effect on individual trees: the growth of epicormic shoots could have been promoted by pruning infected branches or mistletoe removal may have increased light infiltration to the understory. Likewise, transferring biomass from living foliage in the canopy to discrete concentrations of litter on the forest floor may also have had effects. All three of these side-effects are equally likely in the control woodlands, however, as equivalent biomass was removed by haphazardly pruning branches (but leaving mistletoes intact; [28]). Moreover, if these effects did occur across both treatment and control sites, this sudden increase in litter-fall would have boosted litter and availability of litter-dwelling arthropods. By waiting three years after the experimental removal to collect post-treatment data, the influence of these experimental side effects were minimized, previous experimental work demonstrating mistletoe litter decomposes in less than one year [17]. Although Eucalyptus leaves decay more slowly, and some of the thicker branches (of both host and mistletoe) had not decomposed when post-treatment bird surveys were conducted, sham removal ensured volumes of coarse woody debris were comparable between control and treatment sites. Finally, it is possible that the effects noted here relate to differential litter-fall in trees infected with mistletoe compared with otherwise similar but uninfected trees. In previous work, no effects of mistletoe infection on host litter were detected (in terms of either quantity or chemical composition [17]), so I am confident that the experimental treatment affected litter-fall via removing the contribution of mistletoe rather than altering rates of litter-fall from their eucalypt hosts.

Although this is the first empirical study implicating reduced litter-fall in insectivore declines, this mechanism likely reflects a more widespread phenomenon and may be generalizable to insectivore declines more broadly. Rather than affecting distribution patterns directly, habitat structure [5, 9], agricultural intensification [1] and fragment size [42] may operate via changes to the quantity, quality, heterogeneity and persistence of leaf litter. As well as explaining the particular vulnerability of ground-foraging insectivores, this resource-based explanation allows threatening processes at multiple scales to be considered using the common currency of litter. Thus, grazing intensity and frequency, fire, removal of coarse woody debris and clearing of understory shrubs all affect the amount of litter produced and retained within forests and woodlands ([26], and references therein) thereby defining habitat suitability and determining occurrence patterns for several functional groups of animal. In addition to profound changes in prey availability for insectivores, changes in abundance and activity of litter and soil-dwelling arthropods would affect decomposition dynamics and nutrient cycling, especially when coupled with changes in the quality, quantity and temporal variability in litter-fall [43]. By considering ecosystem function, organismal responses and management in terms of litter-fall, seemingly idiosyncratic findings can be considered in functional terms [44], informing directed interventions to achieve lasting improvements to populations and entire ecosystems.
